# {2-[2-(Ethyl­amino)ethyl­imino­meth­yl]-5-methoxy­phenolato}(thio­cyanato-κ*N*)­copper(II)

**DOI:** 10.1107/S1600536810009402

**Published:** 2010-03-17

**Authors:** Yu Zhu

**Affiliations:** a Department of Chemistry, Baicheng Normal College, Baicheng 137000, People’s Republic of China

## Abstract

In the title mononuclear copper(II) complex, [Cu(C_12_H_17_N_2_O_2_)(NCS)], the Cu^II^ atom is four-coordinated by an NNO-donor set of the tridentate Schiff base ligand and the N atom of a terminal thio­cyanate ligand in a slightly distorted square-planar geometry.

## Related literature

For Cu^II^ complexes with Schiff base ligands, see: Dede *et al.* (2009 ▶); Rai (2010 ▶); Rajasekar *et al.* (2010 ▶); Roper *et al.* (1989 ▶). For related structures, see: Adams *et al.* (2003 ▶); Roy & Manassero (2010 ▶).
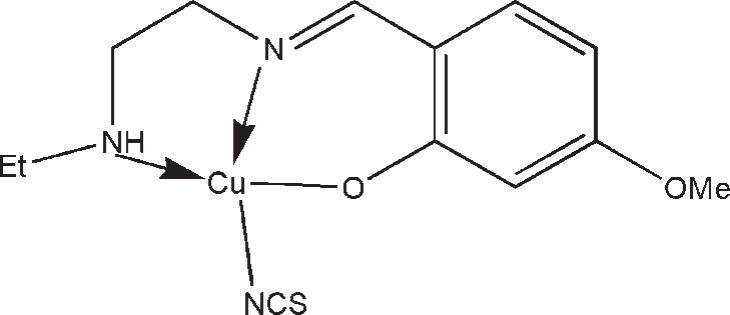


## Experimental

### 

#### Crystal data

[Cu(C_12_H_17_N_2_O_2_)(NCS)]
*M* *_r_* = 342.90
Monoclinic, 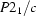

*a* = 12.296 (6) Å
*b* = 10.582 (5) Å
*c* = 12.480 (6) Å
β = 113.810 (7)°
*V* = 1485.7 (12) Å^3^
*Z* = 4
Mo *K*α radiation
μ = 1.61 mm^−1^
*T* = 293 K
0.30 × 0.27 × 0.27 mm

#### Data collection

Bruker SMART CCD area-detector diffractometer
Absorption correction: multi-scan (*SADABS*; Sheldrick, 1996 ▶) *T*_min_ = 0.643, *T*_max_ = 0.670
8523 measured reflections
3282 independent reflections
2123 reflections with *I* > 2σ(*I*)
*R*_int_ = 0.039

#### Refinement

*R*[*F* ^2^ > 2σ(*F* ^2^)] = 0.044
*wR*(*F* ^2^) = 0.129
*S* = 1.00
3282 reflections
183 parameters
H-atom parameters constrained
Δρ_max_ = 0.61 e Å^−3^
Δρ_min_ = −0.34 e Å^−3^

### 

Data collection: *SMART* (Bruker, 1998 ▶); cell refinement: *SAINT* (Bruker, 1998 ▶); data reduction: *SAINT*; program(s) used to solve structure: *SHELXS97* (Sheldrick, 2008 ▶); program(s) used to refine structure: *SHELXL97* (Sheldrick, 2008 ▶); molecular graphics: *SHELXTL* (Sheldrick, 2008 ▶); software used to prepare material for publication: *SHELXTL*.

## Supplementary Material

Crystal structure: contains datablocks global, I. DOI: 10.1107/S1600536810009402/ci5055sup1.cif

Structure factors: contains datablocks I. DOI: 10.1107/S1600536810009402/ci5055Isup2.hkl

Additional supplementary materials:  crystallographic information; 3D view; checkCIF report

## Figures and Tables

**Table 1:** Selected bond lengths (Å)

Cu1—O1	1.817 (3)
Cu1—N1	1.828 (3)
Cu1—N3	1.868 (3)
Cu1—N2	1.912 (4)

**Fig. 1.**
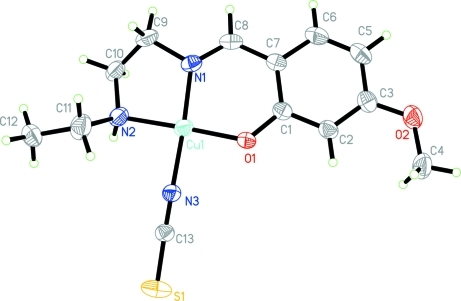
The molecular structure of the title compound, showing the atomic numbering. Displacement ellipsoids are drawn at the 50% probability level.

## supplementary crystallographic information

### Comment

Copper(II) complexes with Schiff base ligands have received much attention in coordination chemistry (Rai, 2010; Roy & Manassero, 2010; Rajasekar *et al.*, 2010; Dede *et al.*, 2009). In the present work, we report the the crystal structure of a new copper(II) complex, the title compound, with the Schiff base ligand 2-[(2-ethylaminoethylimino)methyl]-5-methoxyphenolate.

The Cu^II^ atom in the title complex is four-coordinated by the NNO donor set of the Schiff base ligand, and the N atom of the terminal thiocyanate ligand, in a square-planar geometry. The coordination bond distances (Table 1) are within normal ranges and comparable to those in related complexes (Roper *et al.*, 1989; Adams *et al.*, 2003).

### Experimental

Equimolar quantities (1 mmol each) of 2-hydroxy-4-methoxybenzaldehyde, *N*-ethylethylenediamine, ammonium thiocyanate, and copper nitrate were mixed and stirred in a methanol-acetonitrile (2:1 v/v) solution at room temperature for 3 h. The solution was allowed to evaporate slowly to give needle-shaped single crystals.

### Refinement

H atoms were placed in geometrically idealized positions and constrained to ride on their parent atoms, with C–H = 0.93–0.97 Å, N–H = 0.91 Å, and U_iso_(H) = 1.2U_eq_(C,N) and 1.5U_eq_(C_methyl_).

### Crystal data

**Table:**

[Cu(C_12_H_17_N_2_O_2_)(NCS)]	*F*(000) = 708
*M**_r_* = 342.90	*D*_x_ = 1.533 Mg m^−^^3^
Monoclinic, *P*2_1_/*c*	Mo *K*α radiation, λ = 0.71073 Å
Hall symbol: -P 2ybc	Cell parameters from 2310 reflections
*a* = 12.296 (6) Å	θ = 2.6–25.0°
*b* = 10.582 (5) Å	µ = 1.61 mm^−^^1^
*c* = 12.480 (6) Å	*T* = 293 K
β = 113.810 (7)°	Block cut from needle, blue
*V* = 1485.7 (12) Å^3^	0.30 × 0.27 × 0.27 mm
*Z* = 4	

### Data collection

**Table:**

Bruker SMART CCD area-detector diffractometer	3282 independent reflections
Radiation source: fine-focus sealed tube	2123 reflections with *I* > 2σ(*I*)
graphite	*R*_int_ = 0.039
ω scan	θ_max_ = 27.5°, θ_min_ = 1.8°
Absorption correction: multi-scan (*SADABS*; Sheldrick, 1996)	*h* = −15→15
*T*_min_ = 0.643, *T*_max_ = 0.670	*k* = −13→13
8523 measured reflections	*l* = −16→10

### Refinement

**Table:**

Refinement on *F*^2^	Primary atom site location: structure-invariant direct methods
Least-squares matrix: full	Secondary atom site location: difference Fourier map
*R*[*F*^2^ > 2σ(*F*^2^)] = 0.044	Hydrogen site location: inferred from neighbouring sites
*wR*(*F*^2^) = 0.129	H-atom parameters constrained
*S* = 1.00	*w* = 1/[σ^2^(*F*_o_^2^) + (0.0551*P*)^2^ + 1.5145*P*] where *P* = (*F*_o_^2^ + 2*F*_c_^2^)/3
3282 reflections	(Δ/σ)_max_ = 0.001
183 parameters	Δρ_max_ = 0.61 e Å^−^^3^
0 restraints	Δρ_min_ = −0.34 e Å^−^^3^

### Special details

**Table:**

Geometry. All esds (except the esd in the dihedral angle between two l.s. planes) are estimated using the full covariance matrix. The cell esds are taken into account individually in the estimation of esds in distances, angles and torsion angles; correlations between esds in cell parameters are only used when they are defined by crystal symmetry. An approximate (isotropic) treatment of cell esds is used for estimating esds involving l.s. planes.
Refinement. Refinement of *F*^2^ against ALL reflections. The weighted *R*-factor wR and goodness of fit *S* are based on *F*^2^, conventional *R*-factors *R* are based on *F*, with *F* set to zero for negative *F*^2^. The threshold expression of *F*^2^ > σ(*F*^2^) is used only for calculating *R*-factors(gt) etc. and is not relevant to the choice of reflections for refinement. *R*-factors based on *F*^2^ are statistically about twice as large as those based on *F*, and *R*- factors based on ALL data will be even larger.

### Fractional atomic coordinates and isotropic or equivalent isotropic displacement parameters (Å^2^)

**Table:**

	*x*	*y*	*z*	*U*_iso_*/*U*_eq_	
Cu1	0.55890 (4)	0.38272 (4)	0.94272 (4)	0.04744 (18)	
N1	0.6492 (3)	0.3114 (3)	1.0844 (3)	0.0465 (8)	
N2	0.4301 (3)	0.2831 (3)	0.9449 (3)	0.0596 (9)	
H2N	0.3686	0.3382	0.9287	0.072*	
N3	0.4579 (3)	0.4525 (3)	0.7995 (3)	0.0488 (8)	
O1	0.6770 (2)	0.4836 (3)	0.9392 (2)	0.0543 (7)	
O2	1.0326 (3)	0.7210 (3)	1.0840 (3)	0.0718 (9)	
S1	0.30391 (13)	0.57348 (14)	0.60132 (10)	0.0790 (4)	
C1	0.7807 (3)	0.5063 (4)	1.0246 (3)	0.0460 (9)	
C2	0.8513 (4)	0.5995 (4)	1.0075 (3)	0.0516 (10)	
H2	0.8249	0.6414	0.9360	0.062*	
C3	0.9598 (4)	0.6314 (4)	1.0944 (4)	0.0552 (10)	
C4	0.9978 (5)	0.7842 (5)	0.9760 (4)	0.0858 (17)	
H4A	0.9877	0.7240	0.9152	0.129*	
H4B	1.0578	0.8443	0.9801	0.129*	
H4C	0.9240	0.8276	0.9590	0.129*	
C5	1.0006 (4)	0.5683 (5)	1.2010 (4)	0.0664 (12)	
H5	1.0740	0.5885	1.2598	0.080*	
C6	0.9326 (4)	0.4776 (4)	1.2183 (4)	0.0629 (12)	
H6	0.9605	0.4365	1.2904	0.075*	
C7	0.8217 (4)	0.4421 (4)	1.1327 (3)	0.0480 (9)	
C8	0.7542 (4)	0.3458 (4)	1.1546 (3)	0.0510 (10)	
H8	0.7884	0.3035	1.2258	0.061*	
C9	0.5884 (4)	0.2097 (4)	1.1177 (4)	0.0597 (11)	
H9A	0.6175	0.2042	1.2023	0.072*	
H9B	0.6024	0.1293	1.0880	0.072*	
C10	0.4593 (4)	0.2402 (4)	1.0658 (4)	0.0630 (12)	
H10A	0.4129	0.1660	1.0658	0.076*	
H10B	0.4421	0.3062	1.1106	0.076*	
C11	0.3850 (5)	0.1848 (5)	0.8565 (4)	0.0792 (15)	
H11A	0.4379	0.1126	0.8819	0.095*	
H11B	0.3879	0.2159	0.7845	0.095*	
C12	0.2620 (5)	0.1410 (5)	0.8301 (4)	0.0816 (16)	
H12A	0.2569	0.1124	0.9009	0.122*	
H12B	0.2422	0.0727	0.7748	0.122*	
H12C	0.2074	0.2095	0.7976	0.122*	
C13	0.3932 (4)	0.5018 (4)	0.7173 (3)	0.0459 (9)	

### Atomic displacement parameters (Å^2^)

**Table:**

	*U*^11^	*U*^22^	*U*^33^	*U*^12^	*U*^13^	*U*^23^
Cu1	0.0569 (3)	0.0430 (3)	0.0396 (3)	−0.0019 (2)	0.0166 (2)	0.00224 (19)
N1	0.063 (2)	0.0397 (17)	0.0379 (16)	0.0056 (15)	0.0213 (15)	0.0010 (13)
N2	0.077 (3)	0.0499 (19)	0.0485 (19)	−0.0137 (18)	0.0216 (18)	0.0024 (15)
N3	0.055 (2)	0.0471 (18)	0.0396 (17)	−0.0061 (16)	0.0142 (15)	0.0017 (14)
O1	0.0489 (16)	0.0666 (18)	0.0378 (14)	−0.0067 (14)	0.0073 (12)	0.0121 (12)
O2	0.0598 (19)	0.089 (2)	0.0557 (19)	−0.0252 (18)	0.0123 (15)	−0.0104 (16)
S1	0.0831 (9)	0.1010 (10)	0.0463 (6)	0.0400 (8)	0.0192 (6)	0.0180 (6)
C1	0.046 (2)	0.050 (2)	0.0409 (19)	0.0040 (18)	0.0167 (17)	−0.0023 (17)
C2	0.048 (2)	0.063 (3)	0.040 (2)	−0.002 (2)	0.0142 (17)	−0.0014 (18)
C3	0.052 (2)	0.060 (3)	0.051 (2)	−0.004 (2)	0.0181 (19)	−0.0141 (19)
C4	0.078 (4)	0.106 (4)	0.069 (3)	−0.044 (3)	0.024 (3)	−0.008 (3)
C5	0.051 (3)	0.078 (3)	0.054 (3)	0.002 (2)	0.004 (2)	−0.007 (2)
C6	0.063 (3)	0.068 (3)	0.045 (2)	0.010 (2)	0.008 (2)	0.003 (2)
C7	0.047 (2)	0.050 (2)	0.043 (2)	0.0092 (19)	0.0146 (17)	0.0009 (17)
C8	0.064 (3)	0.048 (2)	0.038 (2)	0.018 (2)	0.0180 (19)	0.0068 (17)
C9	0.087 (3)	0.044 (2)	0.047 (2)	0.001 (2)	0.025 (2)	0.0102 (18)
C10	0.086 (4)	0.052 (2)	0.052 (2)	−0.018 (2)	0.029 (2)	0.002 (2)
C11	0.103 (4)	0.073 (3)	0.065 (3)	−0.030 (3)	0.037 (3)	−0.011 (3)
C12	0.078 (3)	0.093 (4)	0.057 (3)	−0.029 (3)	0.009 (2)	−0.002 (3)
C13	0.054 (2)	0.046 (2)	0.038 (2)	−0.0007 (19)	0.0193 (18)	−0.0025 (17)

### Geometric parameters (Å, °)

**Table:**

Cu1—O1	1.817 (3)	C4—H4B	0.96
Cu1—N1	1.828 (3)	C4—H4C	0.96
Cu1—N3	1.868 (3)	C5—C6	1.346 (6)
Cu1—N2	1.912 (4)	C5—H5	0.93
N1—C8	1.286 (5)	C6—C7	1.402 (6)
N1—C9	1.463 (5)	C6—H6	0.93
N2—C11	1.453 (6)	C7—C8	1.410 (6)
N2—C10	1.474 (5)	C8—H8	0.93
N2—H2N	0.91	C9—C10	1.488 (6)
N3—C13	1.139 (5)	C9—H9A	0.97
O1—C1	1.313 (4)	C9—H9B	0.97
O2—C3	1.346 (5)	C10—H10A	0.97
O2—C4	1.409 (6)	C10—H10B	0.97
S1—C13	1.610 (4)	C11—C12	1.487 (7)
C1—C2	1.386 (6)	C11—H11A	0.97
C1—C7	1.409 (5)	C11—H11B	0.97
C2—C3	1.379 (6)	C12—H12A	0.96
C2—H2	0.93	C12—H12B	0.96
C3—C5	1.389 (6)	C12—H12C	0.96
C4—H4A	0.96		
			
O1—Cu1—N1	94.97 (13)	C3—C5—H5	120.4
O1—Cu1—N3	88.49 (13)	C5—C6—C7	122.9 (4)
N1—Cu1—N3	176.29 (15)	C5—C6—H6	118.5
O1—Cu1—N2	177.40 (14)	C7—C6—H6	118.5
N1—Cu1—N2	86.67 (15)	C6—C7—C1	117.6 (4)
N3—Cu1—N2	89.82 (15)	C6—C7—C8	120.9 (4)
C8—N1—C9	120.1 (3)	C1—C7—C8	121.6 (4)
C8—N1—Cu1	126.4 (3)	N1—C8—C7	125.5 (3)
C9—N1—Cu1	113.4 (3)	N1—C8—H8	117.3
C11—N2—C10	114.7 (3)	C7—C8—H8	117.3
C11—N2—Cu1	116.6 (3)	N1—C9—C10	107.1 (3)
C10—N2—Cu1	108.9 (3)	N1—C9—H9A	110.3
C11—N2—H2N	105.2	C10—C9—H9A	110.3
C10—N2—H2N	105.2	N1—C9—H9B	110.3
Cu1—N2—H2N	105.2	C10—C9—H9B	110.3
C13—N3—Cu1	174.4 (3)	H9A—C9—H9B	108.5
C1—O1—Cu1	127.7 (2)	N2—C10—C9	106.8 (4)
C3—O2—C4	118.0 (3)	N2—C10—H10A	110.4
O1—C1—C2	118.0 (3)	C9—C10—H10A	110.4
O1—C1—C7	122.9 (4)	N2—C10—H10B	110.4
C2—C1—C7	119.1 (4)	C9—C10—H10B	110.4
C3—C2—C1	121.3 (4)	H10A—C10—H10B	108.6
C3—C2—H2	119.3	N2—C11—C12	115.7 (4)
C1—C2—H2	119.3	N2—C11—H11A	108.4
O2—C3—C2	124.5 (4)	C12—C11—H11A	108.4
O2—C3—C5	115.7 (4)	N2—C11—H11B	108.4
C2—C3—C5	119.8 (4)	C12—C11—H11B	108.4
O2—C4—H4A	109.5	H11A—C11—H11B	107.4
O2—C4—H4B	109.5	C11—C12—H12A	109.5
H4A—C4—H4B	109.5	C11—C12—H12B	109.5
O2—C4—H4C	109.5	H12A—C12—H12B	109.5
H4A—C4—H4C	109.5	C11—C12—H12C	109.5
H4B—C4—H4C	109.5	H12A—C12—H12C	109.5
C6—C5—C3	119.3 (4)	H12B—C12—H12C	109.5
C6—C5—H5	120.4	N3—C13—S1	178.8 (4)
